# Larval source management for malaria control in Africa: myths and reality

**DOI:** 10.1186/1475-2875-10-353

**Published:** 2011-12-13

**Authors:** Ulrike Fillinger, Steven W Lindsay

**Affiliations:** 1 Department of Disease Control, London School of Hygiene and Tropical Medicine, Keppel Street, London WC1E 7HT, UK; 2 International Centre of Insect Physiology and Ecology, Thomas Odhiambo Campus, Mbita, Mbita 40305, Kenya

## Abstract

As malaria declines in many African countries there is a growing realization that new interventions need to be added to the front-line vector control tools of long-lasting impregnated nets (LLINs) and indoor residual spraying (IRS) that target adult mosquitoes indoors. Larval source management (LSM) provides the dual benefits of not only reducing numbers of house-entering mosquitoes, but, importantly, also those that bite outdoors. Large-scale LSM was a highly effective method of malaria control in the first half of the twentieth century, but was largely disbanded in favour of IRS with DDT. Today LSM continues to be used in large-scale mosquito abatement programmes in North America and Europe, but has only recently been tested in a few trials of malaria control in contemporary Africa. The results from these trials show that hand-application of larvicides can reduce transmission by 70-90% in settings where mosquito larval habitats are defined but is largely ineffectual where habitats are so extensive that not all of them can be covered on foot, such as areas that experience substantial flooding. Importantly recent evidence shows that LSM can be an effective method of malaria control, especially when combined with LLINs. Nevertheless, there are a number of misconceptions or even myths that hamper the advocacy for LSM by leading international institutions and the uptake of LSM by Malaria Control Programmes. Many argue that LSM is not feasible in Africa due to the high number of small and temporary larval habitats for *Anopheles gambiae *that are difficult to find and treat promptly. Reference is often made to the Ross-Macdonald model to reinforce the view that larval control is ineffective. This paper challenges the notion that LSM cannot be successfully used for malaria control in African transmission settings by highlighting historical and recent successes, discussing its potential in an integrated vector management approach working towards malaria elimination and critically reviewing the most common arguments that are used against the adoption of LSM.

## Background

**Figure 1:**
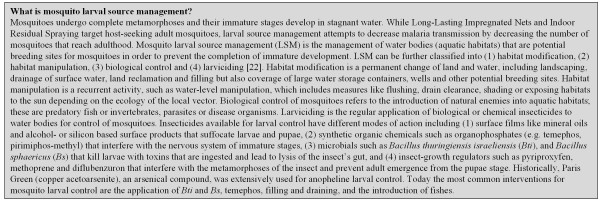
**Summary information on mosquito larval source management**.

The United Nation's Roll Back Malaria decade 2000-2010 has seen an unprecedented increase in the coverage of malaria control interventions. It is a critical time in the history of malaria control in Africa since, for the first time in a generation malaria is declining, at least in some countries [1]. The present global malaria control strategy aims at protecting individuals and communities using long-lasting impregnated nets (LLINs), indoor-residual spraying (IRS) and the prompt and effective treatment of clinical malaria [2]. In order to maintain this momentum and aim for further reductions in malaria transmission, supplementary tools for vector control need to be added to the current arsenal [3]. Since LLINs and IRS are directed against the adult vector population that enters houses, further suppression of transmission could be achieved by targeting the aquatic stages by reducing vector larval habitats, thus attacking both outdoor and indoor biting vectors. This may be particularly important in areas targeted for elimination where malaria foci or 'hot spots' persist [4-9]. At the same time as the global malaria community is considering how to eliminate malaria, the World Health Organization (WHO) is actively promoting Integrated Vector Management (IVM), where multiple interventions are combined to control vector-borne diseases [3,10-15]. Nevertheless, larval source management (LSM, Figure 1), although one of the oldest tools in the fight against malaria remains a largely forgotten and often dismissed intervention for malaria control in Africa [16,17]. Despite the lack of its application in Africa for over half a century, LSM has been the main focus of mosquito control programmes for decades in the United States of America (US), Canada, throughout Europe, Brazil and Singapore [18-20]. In the US larval control has been used for over a century [21]. Today there are 734 named mosquito abatement districts in the US, all employing LSM, which is the 'primary and preferred method of mosquito control in the US, should habitat removal or modification be inadequate' (American Mosquito Control Association, personal communication). LSM is practiced over extensive areas, especially in California and Florida, often controlling mosquitoes that occur on far more prodigious scales than found in Africa. In the largest district, Lee County Florida, the annual budget for mosquito control exceeds $19 M [22], whilst in the Metropolitan Mosquito Abatement District the budget is over $18 M [23]. Despite the scale and success of these operations in developed countries, this activity has been largely ignored by those interested in malaria control, until recently.

This paper challenges the notion that LSM cannot be successfully used for malaria control in African transmission settings by highlighting historical and recent successes, discussing its potential in an IVM approach working towards malaria elimination and critically reviewing the most common arguments that are used against the adoption of LSM. It needs to be emphasized that LSM should not be considered as a stand alone intervention (at least in most circumstances) or replacement for personal protection measures, but an additional tool of IVM. Therefore, this paper does not aim to contrast advantages and disadvantages for LSM with current first line interventions, which can be found elsewhere [24,25], but rather aims to highlight the potential benefits of a neglected tool where applicable.

### Larval source management pre IRS with DDT

In the early twentieth century larviciding and environmental management were the only tools available to contain malaria. The historical literature and more recent reviews of this approach show that anti-larval mosquito control measures were powerful tools against malaria [25,26]. Importantly LSM contributed to all successful eradication efforts and successful vector control programme worldwide [27-33].

The first report of anti-larval measures used for malaria control in Africa was in Freetown, Sierra Leone, in 1812, where there was a law preventing people from allowing stagnant pools which 'generate disease and mosquitoes over the town' [34]. Since then, *Anopheles *larval control has been a central pillar of many successful malaria control programmes worldwide. What is particularly salient, and is relevant to the current push for IVM, is that these programmes all used combinations of vector control tools.

Perhaps the most remarkable achievements with larviciding were the elimination of *Anopheles arabiensis *[35], a member of the *An. gambiae *complex, from Brazil [28] and Egypt [27]. In the 1930s, *An. arabiensis*, a major vector of malaria in Africa, was introduced accidentally into Brazil resulting in an epidemic that killed thousands of people and turned the countryside into a wilderness [28,33]. Most remarkably, a larval control programme run with military precision was able to eradicate *An. arabiensis *within 2-3 years, under-budget and on schedule. The common larval habitats in Brazil resembled those found in many African settings [36], in a climate similar to parts of Africa where malaria is endemic [37]. Similarly, when *An. arabiensis *invaded Egypt in 1942, the vector was eliminated using larval control within a staggeringly short time of 6 months [27].

These vectors have also been successfully controlled in the heartland of malaria: in Africa. Malaria was a major threat to the economic success of the copper mines in Zambia in the first half of the twentieth century. An integrated malaria vector control programme, primarily based on attacking the larval stages of malaria vectors by environmental management [29] resulted in a 97% reduction of annual malaria incidence from 514/1,000 in 1929/1930 to 16/1,000 in 1949/1950. Similarly, overall mortality fell by 88% from 32/1,000/year to 4/1,000/year. Drainage of breeding sites along the Nigerian coast led to a 77% decrease of malaria incidence from 130/1,000/year in 1942 to 30/1,000/year in 1943. Interestingly, only the addition of environmental management to established interventions like quinine treatment and personal protection measures led to such significant decreases in malaria whilst there was hardly any impact before LSM was introduced [38]. LSM was not limited to Africa and was most successfully employed for malaria control in South East Asia, particularly in Malaysia and Indonesia [26,39-41].

### The fate of LSM after IRS with DDT

Malaria eradication with IRS using DDT sounded the death knell for many effective control methods, including LSM. LSM is based on a sound understanding of the local ecology of malaria in an area. It is also complex and requires strong management [42-45]. The rush for malaria eradication with IRS and DDT represented, at that time, a simple fix that could be used anywhere unlike LSM. The result was eloquently captured by Socrates Litsios [46]:

'With the arrival of DDT the detailed understanding that had built up in the course of tens of thousands of studies was put aside and a monolithic strategy took hold. With victory in sight, there was no need for further study. Today, when victory seems far away, there is a risk that what was learned before DDT arrived will be forgotten'.

The failure of the global malaria eradication programme had repercussions that put vector control research in the doldrums for several decades. The resurgence of interest in vector control coincided with the renewed efforts to accelerate malaria control in Africa and the development of insecticide-treated bed nets in the 1980s [47,48] as a practical control tool, but the focus of research from then on focused heavily on attacking vectors indoors with insecticides, almost excluding alternative approaches [49]. However, over the past decade, there have been opportunities for diversification and a reappraisal of many forms of control, including LSM [2].

### Recent evidence for the potential of LSM in Africa

Recent field evaluations (Table 1) under various eco-epidemiological conditions in Africa showed that: (1) hand-applied larviciding reduced transmission by 70-90% where the majority of aquatic mosquito larval habitats were defined and aquatic surface areas not too extensive [43,50-52] and (2) that the addition of larviciding with LLINs resulted in greater gains than could be achieved by using LLINs alone [52,53]. The cautionary note is that hand-application of larvicides was not effective in areas with very extensive water bodies such as the floodplains of the large river system in the middle reaches of the Gambia River [54]. But as we make progress towards malaria elimination, it may be that persistent malarious areas can be effectively controlled by aerial application of larvicides, which would be best suited for the treatment of extensive flood plains and irrigation systems [19,22,23,55]. Although this method of application is expensive, if it results in elimination, these costs may be justifiable, in the same way as aerial application was in the Onchocerciasis Control Programme in West Africa [56,57].

**Table 1:** Recent trials of larvicides against malaria in Africa

Study site	Ecosystem	Reduction in			Date of trial
			
		Late instar *Anopheles *larval density	*Anopheles gambiae s.l*. adult density	Malaria infection	
Semi-arid ecosystems, Eritrea [50]	Desert fringe	Significant reduction	Significant reduction	-	Not reported

Lake Victoria, Kenya [51]	Rural, high population density	99% (97.5-99.4%)	91.5% (91.4-91.6%)	-	Jul 2001-Sep 2005

Western Highlands, Kenya [52]	Rural, Highlands	91% (87-95%)	86% (80-88%)	56% (18-77%)	Feb 2004-Jan 2007

Dar es Salaam, Tanzania [43,53]	Urban	Not done^1^	34.5% (19.1-46.7%)^2^	72% (20-90%)	Apr 2005-May 2007

Middle reaches of the Gambia River, The Gambia [54]	Floodplains	73-99%^3^	No impact	No protection	Jul 2005-Nov 2007

^1^Larval density was not measured but proportion of habitats that contained late *Anopheles *larvae. There was a 96.5% reduction in *Anopheles gambiae *larval habitat abundance in year 1 as compared to the same time period pre-intervention and non-intervention sites [43]

^2^represents overall reduction in year 1 of intervention but late start during rainy season and operational challenges responsible for relative small reduction overall, the dry season larviciding in from July to September reduced transmission by 67% compared with the same time period pre-intervention and non-intervention sites

^3^Reduction in sites containing larvae compared with contemporary controls

### The benefits and role of larval source management in malaria control and elimination

*Anopheles *larvae are 'sitting ducks'; they are relatively immobile and often readily accessible. By targeting the larval stages, mosquitoes are killed '*whole sale*' before they disperse to human habitations. Mosquito larvae, unlike adults, cannot change their habitat to avoid control activities [58].

The elimination of aquatic habitats close to human habitations by environmental modifications and manipulations, where possible, can provide long-term and cost-effective solutions. Once a habitat is gone it does not produce any flying and biting mosquitoes [29,39]. This is particularly true in urban areas where drainage of aquatic habitats can be incorporated into on-going town or city development plans [59,60]. In many cases these costs will be paid outside the health sector. In places where habitats cannot be eliminated, a number of very effective larvicides are available that reduce mosquito production rapidly. There are a broad range of effective formulations that have been developed for anopheline control [24,61,62]. The diverse family of larvicides provide a wide range of modes of actions against *Anopheles *larvae including microbials that lyse the gut epithelium, insect growth regulators that prevent the larvae developing into adults, synthetic or botanical toxins that directly interfere with the insects' metabolism and monolayers that lead to suffocation of larvae. Today's larvicides are environmentally acceptable with minimal or no effect on non-target invertebrate populations, aquatic ecosystems, beneficiary insects, fish, birds, and mammals, including humans. Larviciding requires no substantial change in human behaviour or the management of key resources such as water and land, and skills for larviciding can be similarly acquired as those for IRS [43,52,63,64].

LSM is a well-established strategy, with large-scale programmes worldwide [18-20,22,23,65]. There are many National Malaria Control Programmes in Africa that would be in the position to incorporate, or have already incorporated, LSM in their development agenda [66-72]. The tool is ready to use [19,21,25,43] without any further research required. Obviously, locally appropriate implementation systems need to be developed on an individual basis for each programme, taking local structures and administrative systems into account and adapted to local eco-epidemiological conditions [28,43,73-75]. Sustainable LSM programmes need time for implementation staff and institutions to develop, pilot, refine and stabilize locally-appropriate, effective and sustainable procedures and institutional structures [42,45,76,77]. The scale at which LSM is applied depends on the local ecology, institutional structures and financial support.

Over the past decade interest in LSM by the international scientific community has grown and its potential has been demonstrated for contemporary Africa (Table 1). As a consequence, LSM has been included in the latest Global Malaria Action Plan of the Roll Back Malaria Partnership. The document outlines that in areas where malaria transmission is low to moderate, seasonal or focal the integration of LSM can be appropriate. It is viewed as a targeted approach in addition to LLINs and/or IRS. The added value of LSM is especially anticipated during the phase of 'sustained control' (as opposed to 'scale-up-for-impact') [2]. This is echoed in the Global Malaria Programme for Malaria Elimination where it is stated that 'larviciding may play an important supportive or even leading role in some special settings' [12]. It has been recognized that malaria control interventions must take more account of the mosquito behaviour and the potential adaptability of mosquitoes [49]. Such adaptability has been observed even during historical control interventions [78-81]. Recent publications also convincingly demonstrate that as malaria declines in many African countries, driven down (partly) by the use of LLINs and IRS, outdoor biting is becoming a more important feature of malaria transmission [82-85] with the more exophilic *An. arabiensis *increasing in importance as vectors [86-89]. Griffin and colleagues [90] recently presented strong evidence that outdoor biting defines the limit of what is achievable with LLINs and IRS. LSM is one of the few strategies effective against outdoor biting vectors.

Insecticides used for the control of vectors indoors are limited at present to four different classes: organochlorines, pyrethroids, organophosphates and carbamates. The wide diversity of insecticides used for larval control, many of which are not used for adult control, represents an important opportunity to maintain the longevity of insecticides for adult control, especially if combined with environmental management. This is particularly relevant today when resistance to pyrethroids, used for treating bed nets and IRS, is threatening the effectiveness of control programmes across Africa [91,92]. There is also an obligation to replace DDT with other insecticides [93], further restricting our ability to deal with resistance. Last, but not least, LSM could have a role to play in malaria eradication where persistent malaria 'hot spots' remain, after the application of existing tools directed at indoor-feeding vectors.

### Why is LSM not considered on par with LLINs and IRS?

The question posed here is why, with all the historical and recent evidence, LSM is not considered 'on par with LLINs and IRS' [2] today? There are a number of reasons for this, some understandable, some plainly wrong.

#### Evidence of efficacy

Interventions against malaria are typically evaluated by measuring a decline in malaria morbidity and mortality. This is usually done by randomly allocating the test intervention and a placebo of current intervention at the level of the individual, household or cluster of houses. The randomized controlled trial (RCT) has become the standard tool for evaluating interventions [94,95]. Since LSM needs to be applied over large-scales of many square kilometres it is impracticable or prohibitively expensive to carry out a large-scale RCT. Consequently, there will never be the same degree of proof that LSM is effective, as is available with interventions that are randomized by individual or household, such as with LLINs [96]. In this context, LSM is very similar to that of IRS where the main evidence of efficacy is also based on historical accounts and where there are few high-quality trials to measure their impact [97]. Yet today IRS campaigns are common in Africa, whilst there are few LSM programmes in operation [1]. Nonetheless, ultimately the value of an intervention depends on its effectiveness when operated through control programmes and the scalability of the intervention. Although LSM can be scaled up [19,22,65] to date larval control programmes in sub-Saharan Africa have never covered very large areas and populations.

#### Biological myths

During the DDT era and the subsequent production of entomologists who focused on attacking the vectors indoors some common misconceptions have become dogma and reinforced the view that larval control is ineffective. As recently as 2000, the WHO expert committee on malaria control did not consider LSM in their packages of interventions [98]. One of the reasons for this was the Ross-Macdonald model [99] that defined one of the keystones of the IRS DDT era. According to this model the greatest reductions in malaria transmission can be achieved by reducing the longevity of the vector population. This was best achieved by killing the vectors indoors, which would result in a reduction of survival of the vector population, as well as reducing vector numbers, rather than attacking the aquatic stages where survival would not be affected. Based on this model, the original assumptions made in the first eradication campaigns were very simplistic [100]. Nevertheless, a point which the rational of MacDonald [101] and Garrett-Jones [100] missed is that it is equally important to assess how easy parameters are to change as it is to assess the relative magnitude of the impact that changing those parameters delivers. More current models show that although killing adult mosquitoes has the highest benefit in reducing malaria transmission, there are limits on increasing adult mosquito mortality above a certain threshold primarily due to changing mosquito behaviour and physiology and the effects of reducing adult emergence is multiplicative and has an even greater effect on R_0 _than reducing survival alone [90,102]. Some models highlight the potential benefit of adding LSM to IVM programmes [103,104]. Several authors have convincingly shown that the limitations of LLINs/ITNs and IRS are largely defined by mosquitoes avoiding them by feeding or resting outdoors and/or at earlier hours and by developing insecticide resistance [82,83,85-89,91,92,105,106]. These concerns can be reduced if LSM is combined with indoor vector control tools. Moreover, recent research also suggests that LSM will not only reduce the number of adult vectors, it may also increase the difficulty an adult female has locating a site to lay her eggs, extending the gonotrophic cycle, and reducing transmission risk [104,107,108].

Many argue that LSM is not feasible in Africa due to the high number of small and temporary larval habitats for *An. gambiae *that are difficult to find and treat promptly, that the delivery of larvicides to very small habitats (e.g. cattle hoof prints) is difficult, and environmental management targets primarily larger, permanent water bodies, which are not typically anopheline habitats and therefore contribute little to malaria control [16,17]. Recent studies show that these assertions are incorrect in many areas of sub-Saharan Africa with stable malaria transmission. Importantly, the widely feared small and temporary habitats contribute little to the overall production of larvae and adults throughout the year [109-112]. For example a study of potential mosquito larval habitats in a 400 km^2 ^area in The Gambia during the rainy season [113] found only 50 puddles or tyres tracks containing water of which 46% had anophelines. This contrasted with 413 ricefields of which 66% had anopheline larvae. Similarly in rural site in western Kenya borrow pits accounted for 60-78% of the total pupal productivity [109] and in the western Kenya highlands puddles, though most productive when present, were the most unstable habitats and accounted only for 5% of all aquatic habitats in the study area whilst permanent drains accounted for 72% [106]. Importantly, today malaria in Africa has become much associated with agricultural development, both in rural and urban settings due to the increasing use of irrigation leading to an increasing number of anopheline habitats [114-120]. Whilst covering all available habitats in the target area at the time of application is aimed for, missing out on a few small, transient habitats that might be overlooked or hard to access is not going to jeopardize the impact of the intervention. It is these larger, semi-permanent and permanent habitats that are often man-made [110,113,121-123], that are static and accessible that are at greater or at least equal risk of being colonized by anophelines than small ones, and these larger sites are available for extended periods of time and are therefore responsible for endemic malaria transmission [106,110,124,125].

Utilization of state of the art mapping tools like Geographical Positioning Systems, Geographical Information Systems and remotely-sensed imagery combined with modern communication tools increases the operational efficiency of disease control interventions, and are successfully used for mosquito vector surveillance and control for example in Australia, Singapore, Nicaragua and the US [126]. GIS was introduced in the operational malaria control programme in South Africa as early as 1990 and is since successfully used for a large number of applications including monitoring of malaria cases and coverage of vector control interventions [127,128]. This is a technology whose application cannot be underestimated with regard to LSM. In previous times, mapping and reconnaissance of larval habitats were necessarily laborious and done by pencil and paper mapping; now, superior technologies allow for mapping and modelling of landscapes to facilitate tremendously the location and treatment of larval habitats; and the retreatment and inspection, when necessary [19,43,55,64,76,129].

#### Management and costs of LSM

The current strategy of LSM with larvicides is to treat all available larval habitats [43,52,54,130]. Some argue for a more spatially targeted approach [131,132] to apply larvicides only at the most productive habitats [133]. At present though we still lack scalable field methods for determining which habitat subsets are the productive ones. In fact to date no published evidence exists that shows that accurately determining where malaria vectors will develop is possible [106,124,134,135]. There is both spatial and temporal variation in the distribution of *Anopheles *larvae. Whilst some types of habitats are more likely than others to have aquatic stages [106,109,113,124,136], this is not sufficiently refined for spray personnel to be able to identify and target only these high-risk habitats. Most importantly, when it comes to the implementation of LSM, treatment of all sites is much easier for field personnel since this requires minimum decision making and is, therefore, less prone to mistakes [43,134]. However, several models have been developed recently to predict mosquito larval habitats location and productive potential, so in future it may well be possible to target interventions more effectively [137,138]. Any benefit of targeting larval habitats at specific times of the year needs to be proven, but may work well when LSM is part of an IVM package of interventions [52]. Thus, in the future, LSM may be targeted in space, when 'hotspots' of transmission have been identified, or in time, to restrict biting densities at certain times of the year [75]. In both cases the scale of the intervention would be considerably smaller than the routine application of blanket larviciding.

Another concern is the application frequency of larvicides. At present microbial larvicides are generally applied weekly to all potential sites [43]. Whilst larvicides with greater residual activity would be beneficial for treating permanent habitats [139], it is important to note that they are not necessarily the panacea they might appear to be since during periods of rain new potential mosquito larval habitats can appear and larvae can develop into adults before the next round of application. Thus where sites are dynamic, weekly application is effective because new sites are treated promptly and it is simpler because the people who apply the larvicide become familiar with their treatment area and the weekly cycle of activity.

Overall, targeting interventions in space and time as well as the use of more residual larvicides will only reduce costs if proven to be equally effective than blanket application and if the increased management effort for decision making does not outweigh the larvicide costs [140]. Nonetheless, substantial reductions in long-term costs may be made if larviciding is combined with environmental management. A recent study in Dar es Salaam demonstrated that simply by improving drainage in drains would reduce larval breeding by 40% [59]. Since malaria is a problem created by surface water, it is still surprising that engineers are rarely engaged in malaria control [3] since there are many simple and effective engineering solutions to reduce mosquito larval habitats [141].

A frequent critique is that larviciding is too labour intensive for the reasons outlined above. It needs labour intensive management systems for application, surveillance and evaluation, which are expensive and prone to failure [42,45,142]. There is no local capacity in country to implement and evaluate LSM, and it hinders the delivery of other malaria control initiatives. Whilst it is true that LSM requires a large number of personnel, Africa has a large pool of people who could be gainfully employed in large control programmes. This should be viewed as an opportunity rather than an impediment. Similarly, locally appropriate implementation systems take time to be developed and to address initial challenges and failures [42,45]. This is common to all vector control programmes, not just ones using LSM. It may be considered appropriate to consider the role of vector control programmes for the creation of employment in resource-poor communities, which under most circumstances lack other income-generating opportunities. The involvement and payment, therefore, of community members in local (supervised and monitored) vector control activities could therefore contribute to reduction of disease burden, through the reduction of vectors and, indirectly, by improvement of the local socio-economic situation [10,74,75].

LSM has several aspects that are significantly more sustainable than IRS and LLINs since highly effective tools other than larviciding can be applied by local communities without dependency of high recurrent costs [143-146]. The need for local adaptation and skills should be seen as an opportunity creating self-empowerment for health control, which is one of the objectives of the WHO's IVM strategy [13].

A recent analysis of three LSM programmes of different sizes and ecological settings in Africa showed the cost per person protected each year ranged from US$0.94 to US$2.50 [75]. This compares favourably with IRS (range from various African settings US$0.88-4.94, [147]) or LLINs (range for LLINs costing US$5 and assumed to last three years US$1.48-2.64 [148]), suggesting that LSM presents a viable and cost effective malaria control tool that can complement existing malaria control methods in many settings across Africa. With the move towards elimination there is a need to scale-up use of existing tools and use additional cost effective tools to reach that goal. Africa lacks local capacity in trained entomologists and ecologists [49,74]. Yet whether it is malaria elimination or IVM or both, capacity will need to be increased. Human resources need to be improved to ensure that any improved control can be sustained [74,149,150].

## Conclusion

LSM is an important suite of tools for including in IVM packages that will ensure more effective control of malaria. LSM can, as a secondary tool, synergize with primary interventions such as LLINs or IRS. LSM is not a stand-alone intervention but should, where practicable, be integrated with established interventions directed at adult mosquitoes. However, it is not an intervention that can be applied cost-effectively everywhere and specific settings where aquatic habitats are too extensive will be unsuitable, unless aerial application of larvicides is undertaken. This statement simply reinforces the adage that "all malaria is local" and those local conditions need to be considered for all types of interventions, not just LSM.

Mosquito larval control will work best and be most cost-effective in areas where larval habitats are well-defined possibly seasonal or relatively few, where habitats are accessible by ground crews, and in cooler parts of Africa where larval development is prolonged. These conditions occur frequently, even in sub-Saharan Africa, and thus this method can be an effective tool for malaria control in selected eco-epidemiological conditions such as areas of low to medium transmission intensity, areas of focal transmission or epidemic prone areas. Such conditions are common in urban environments, desert fringe communities, highland settlements and rural areas with high population densities.

It is not a strategy for country-wide application, and should not be the primary tool selected in areas of intensive transmission. Nevertheless, LSM has the potential to be integrated into control programmes after LLINs or IRS have reduced transmission to moderate or low levels of transmission and therefore should be considered in the consolidation phase of control and elimination programmes where it can be targeted in space and time. LSM will further reduce transmission, in a synergistic fashion, and help manage insecticide resistance.

## Competing interests

The authors declare that they have no competing interests.

## Authors' contributions

UF and SWL collated the material for this publication and drafted the manuscript. Both authors read and approved the final manuscript.
